# Complications Related to Metal-on-Metal Articulation in Trapeziometacarpal Joint Total Joint Arthroplasty

**DOI:** 10.3390/jfb6020318

**Published:** 2015-05-25

**Authors:** Christina Frølich, Torben Bæk Hansen

**Affiliations:** University Clinic for Hand, Hip and Knee Surgery, Regional Hospital Holstebro, Aarhus University, Lægårdvej 12, DK-7500 Holstebro, Denmark; E-Mail: chrifroe@rm.dk

**Keywords:** metal-on-metal articulation, trapeziometacarpal joint osteoarthritis, trapeziometacarpal joint total joint arthroplasty, metal hypersensitivity, pseudo tumor

## Abstract

Adverse reactions to metal-on-metal (MoM) prostheses are well known from total hip joint resurfacing arthroplasty with elevated serum chrome or cobalt, pain and pseudo tumor formation. It may, however, also be seen after total joint replacement of the trapeziometacarpal joint using MoM articulation, and we present two cases of failure of MoM prostheses due to elevated metal-serum levels in one case and pseudo tumor formation in another case. Furthermore, we suggest a diagnostic algorithm for joint pain after MoM trapeziometacarpal joint replacement based on published experiences from MoM hip prostheses and adverse reactions to metal.

## 1. Introduction

The trapeziometacarpal joint is a common site for osteoarthritis, especially in females in their fifth to seventh decade of life, and typically presents with pain, loss of function and reduced pinch and grip strength. A range of treatment options exists both non-operatives, e.g., splints and medication, and operatives, e.g., trapeziectomy with or without ligament reconstruction and tendon interposition, arthrodesis and total joint prosthesis [1]. No one operative treatment seems however to be superior to all others in the treatment of osteoarthritis of the trapeziometacarpal joint, and the choice of treatment is primarily dependent on the surgeon’s preference, the patient’s age and the severity of the symptoms [1]. 

Total joint prosthesis however may have some advantages. A failed prosthesis can be replaced by almost any of the other operative treatment choices, and Kaszap *et al.* [2] described that function after secondary trapeziectomy is as good as after a primary trapeziectomy. Total joint prostheses for the trapeziometacarpal joint were introduced in the 1970s and 1980s, in the beginning with silicone and later with components of metal and polyethylene [3]. The design resembles that of hip prostheses with a ball and socket joint, and during the last ten years trapeziometacarpal prostheses with a metal-on-metal (MoM) ball and socket design have been used [4] to surpass possible problems with aseptic loosening of polyethylene-on-metal (MoP) prostheses. Total MoM joint prosthesis has shown good short-term results with faster rehabilitation, better strength and less pain than trapeziectomy [5], and in longer follow up has shown continued good results regarding strength, pain relief and mobility [4].

MoM hip prostheses have in recent years been the focus of intensive research, due to the release of metal particles into the periprosthetic tissue and blood stream. The release of metal debris is seen following wear and corrosion of the implant components, releasing particles into the periprosthetic tissues [6]. MoM prostheses are primarily composed of cobalt, chrome and nickel [7], and these metals are found in higher concentrations in the periprosthetic tissue and in the blood in persons with MoM hip prostheses than in persons with MoP prostheses [8,9]. The release of metal ions can result in both local and systemic reactions, e.g., deafness, cardiomyopathy and carcinogenic effects [7,10]. Locally fluid collection and mass formation is seen resulting in osteolysis, tissue necrosis and pseudo tumor formation [10,11]. Pseudo tumors or acute lymphocytic vasculitis associated lesion (ALVAL) are soft tissue masses around a MoM joint prosthesis found not to be infectious or malignant [12]. Increased concentrations of serum metal and metal hypersensitivity have been suspected to lead to the formation of pseudo tumors [13]. Chromium, cobalt and nickel are all known allergenic, and high concentrations can accumulate in the body leading to sensitization and a type IV T-cell mediated hypersensitivity reaction [14]. However the cause and effect relationship between MoM prostheses and metal allergy are not clearly established. 

We present two case stories of patients with MoM trapeziometacarpal prosthesis revised due to MoM related complications with pseudo tumor formation in one case and symptomatic elevated metal ion levels in the other. 

## 2. Results 

### Case Presentation

#### Case 1

A 53-years old female was treated with an Elektra MoM cementless trapeziometacarpal prosthesis (Small Bone Innovations Inc., Les Bruyères, France) for osteoarthritis in her right trapeziometacarpal joint. The Elektra prosthesis is a titanium hydroxyapatite coated metacarpal pressfit stem that comes in four sizes (extra small, small, medium and large—in this case we used size medium) and a modular chrome-cobalt (CoCrMo ISO 5832-12 [15]) neck and head. The cup is a one size bi-metal screw cup (the first generation was made of chrome-cobalt) with a hydroxyapatite-coated titanium cup and a chrome-cobalt press fitted insert (CoCrMo ISO 5832-12) for articulation.

**Figure 1:**
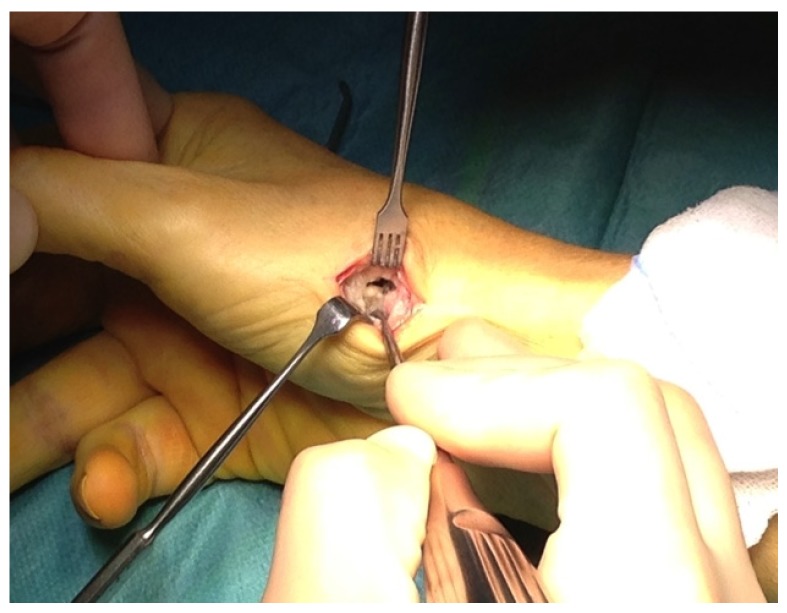
Pseudotumor surrounding the prosthesis.

**Figure 2:**
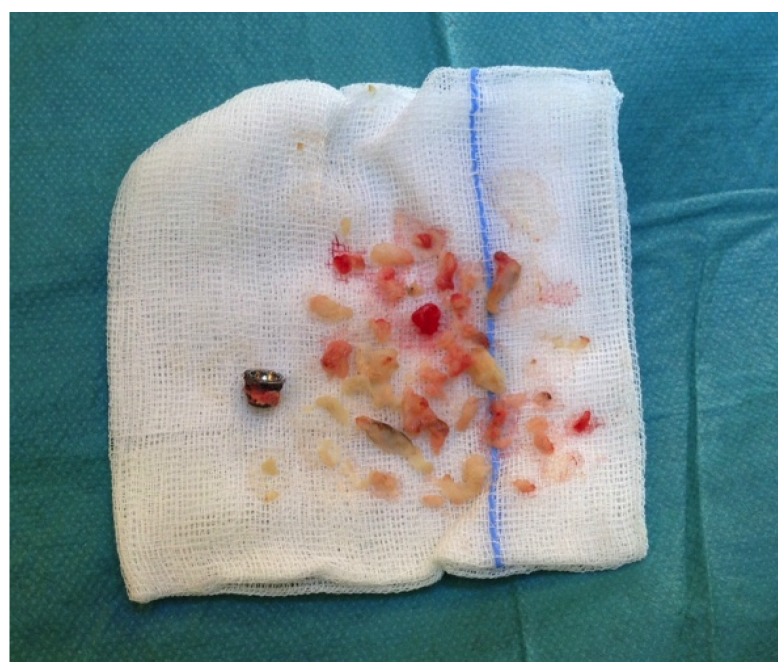
Pseudotumor material and cup removed during surgery.

The patient was pain free and with good mobility after 3 months and returned to heavy hand demanding job as a health care technician. After almost 3 years she presented with pain in the thumb at activity and swelling at the joint. Radiographs revealed bone resorption at the base of the 1. metacarpal but no change in the position of the stem and cup. Serum-cobalt and serum-chrome were found to be normal (<10 nmol/L), but revision surgery was performed on suspicion of pseudo tumor formation. A cystic pseudo tumor surrounding the prosthesis was found at surgery (Figure 1), and removed along with the cup (Figure 2). Both the cup and the stem were found with osseous integration and without signs of implant loosening. A trapeziectomy was performed together with an abductor pollicis longus-suspension arthroplasty. Pseudo tumor material was sent to culture and microscopy, showing no signs of infection but histologically with signs of aseptic necrosis and fibrosis characteristic for pseudo tumor formation. After surgery the patient informed the surgeon, that she had recently been diagnosed with nickel allergy.

At follow up after 12 months the patient was pain free, with good mobility of the thumb and had started working again. 

#### Case 2

**Figure 3:**
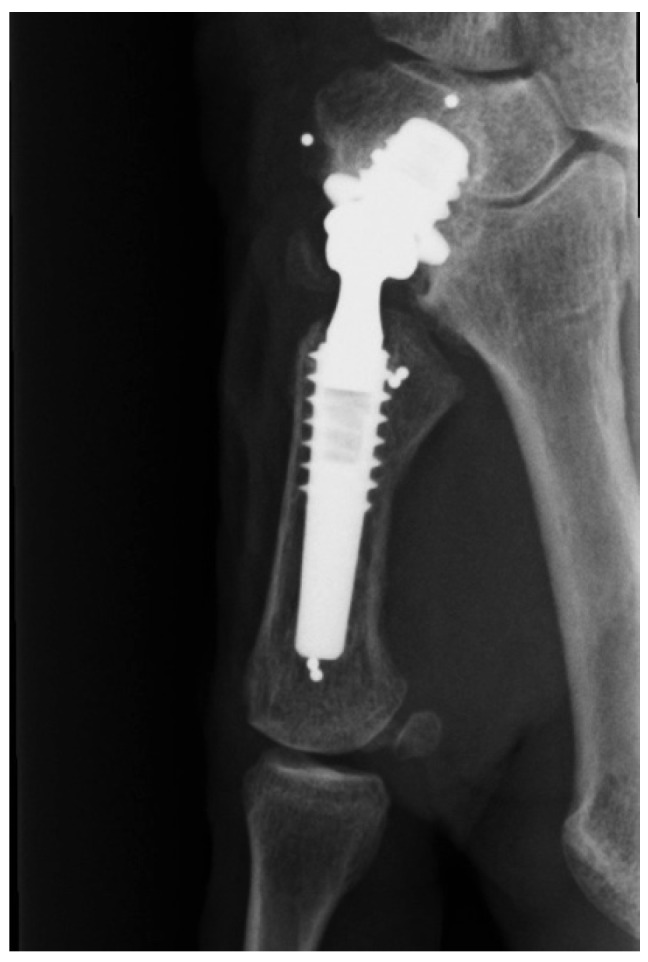
Radiograph without signs of implant failure after 29 months.

**Figure 4:**
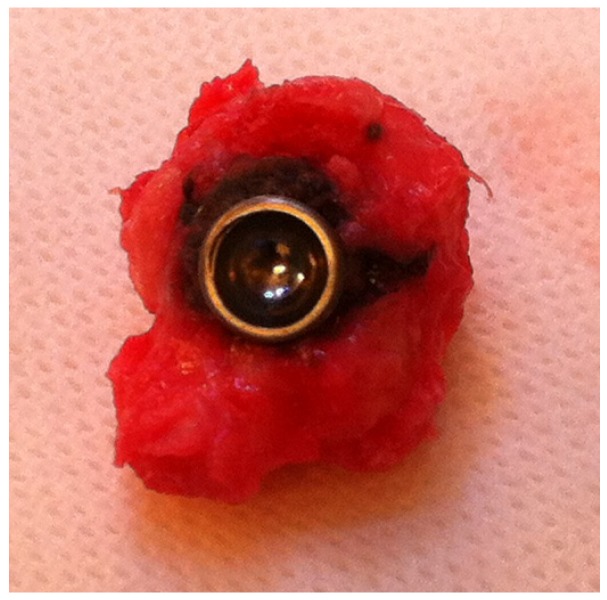
Implant showing signs of wear at the dorso-radial rim, but only slight discoloration of the soft tissue.

A 74-years old female was treated with a trapeziometacarpal MoM prosthesis due to osteoarthritis. The prosthesis used was a cementless Motec titanium Bonit^®^ coated screw cup (Swemac AB, Linköbing, Sweden) with a chrome-cobalt insert (CoCrMo ISO 5832-12) for articulation. The cup comes in three sizes (7.0, 8.5, 10.0) and a size 8.5 was used. The cup was combined with a Motec titanium Bonit^®^ coated metacarpal screw stem with a modular chrome-cobalt (CoCrMo ISO 5832-12) neck and head. The stem comes in four sizes and a size medium was used. Follow up at 29 months showed good function of the prosthesis with a DASH score of 3 (Disabilities of the Arm Shoulder and Hand score, range 0–100 where 0 represents no symptoms) compared to a pre-operative DASH score of 29. Conventional X-rays was found without radiolucent zones, bone resorption, change in position or any other signs of loosening (Figure 3). Blood samples showed elevated S-cobalt 25.4 nmol/L (normal < 10 nmol/L) and S-chrome 25.5 nmol/L (normal < 10 nmol/L). Metal ion levels were measured again three times with 3 months interval, and were found to be continuously at the same level. The patient had some skin rash problems, and insisted on having the prosthesis removed due to the elevated metal serum levels. During surgery the prosthesis showed clear signs of wear at the dorso-radial rim and the preprosthetic tissue was seen to be slightly discolored (Figure 4). However, there were no signs of pseudo tumor formation or discoloration of the bone, and the implant was found well fixated to the bone.

After surgery the metal ion levels rapidly returned to normal values, and the problems with skin rashes disappeared. 

## 3. Discussion

These two case stories represent clinical problems due to metal debris in MoM trapeziometacarpal prosthesis years after the primary surgery with pseudotumor formation or metal sensitivity. 

The prevalence of pseudo tumor formation in MOM hip replacement ranges from 2%–69% [16,17]. The very large difference in prevalence may be due to the heterogenecity, with studies based on hip revision material, symptomatic patients or asymptomatic patients. A wide range of serum metal ion levels has been reported, and some have reported a correlation with pseudo tumor formation [14,18,19], however others have found no correlation between pseudo tumor formation and serum metal ion levels [6,20,21,22].

Metal hypersensitivity has also been investigated. Hallab *et al.* [23] showed a correlation between hypersensitivity and the level of nickel and cobalt debris in MoM hip patients, but did not report the incidence of pseudotumors. Kwon *et al.* [16] and Hasegawa *et al.* [24], however, showed no correlation between metal hypersensitivity and pseudotumor formation. 

Failure of MoM trapeziometacarpal arthroplasties due to adverse reactions to metal debris has been reported by Smith *et al.* [25] and Regnard [4]. Metal release from MoM prostheses may give rise to two key responses; a non-specific machrophage mediated granulomatous response and a specific type-IV T-cell mediated hypersensitivity reaction [11,26]. The non-specific response is associated with a foreign-body reaction, and can be due to metal debris, and has also been reported in MoP hips. The specific type-IV reaction is associated with hypersensitivity and lymphocytic infiltration. Lohmann *et al.* [22] reported a possible dose-depended response and an increase in lymphocyte reactivity when exposed to increased metal debris. The non-specific response was found to be related to low metal concentration in the periprosthetic tissue, and showed no correlation with increased metal exposure. These two different reactions can be representatives of two different causes of failure, and patients with a low threshold can develop metal hypersensitivity leading to failure of the prosthesis. Unfortunately we did not test the two patients for a hypersensitivity reaction, as focus was not on metal related complications at the time of the operation. None of the patients had loose implants, and the metal reaction was not based on metal rubbing the surrounding bone due to movements of a loose implant. The two cases had different implants with a different design in the part that is bony fixated, but both implants are a ball and socket articulation using the same CoCrMo ISO 5832-12 steel articulation. So in our view the source of metal debris is the same in both implant types, and caused by the ball and socket MoM articulation. 

Metal debris can trigger a local inflammatory response, resulting in aseptic osteolysis, tissue necrosis and fluid collection [11,27,28]. This may lead to pseudo tumor formation and ultimately to failure of the prosthesis. Patients typically presents with pain of the joint, decreased function or swelling around the joint. Bisschop *et al.* [19] found that in hip arthroplasty 72.5% of pseudo tumors were asymptomatic, and symptoms were associated with larger pseudo tumors, which also were reported by Hart *et al.* [29]. The natural progression of pseudo tumors is however unknown. Almousa *et al.* [13] followed asymptomatic patients with pseudo tumors and found an increase in size in 40% and they disappeared in 20% of the cases. Primarily small pseudo tumors disappeared. So it is important to follow patients with pseudo tumor formation, as revision surgery after the formation is associated with poorer outcome due to soft tissue necrosis surrounding the prosthesis [30].

The predictors for formation of pseudo tumors are primarily studied in MoM hip arthroplasty. Risk factors include female sex, metal hypersensitivity, component positioning and elevated metal ion levels [10]. The correlation between metal ion levels and pseudo tumor formation remains however unclear; some studies describes a positive correlation [14,19], but serum-metal should never stand alone in the assessment of a patient with a painful joint following joint replacement with MoM implants—especially in trapeziometacarpal joint arthroplasties as these are smaller, and the metal release may be less than in a MoM hip arthroplasty not leading to detectable serum chrome or cobalt values. Hansen *et al.* [31] found that 80% of patients with trapeziometacarpal arthroplasties had normal serum cobalt and chrome, however one of the cases presented in this paper had normal metal values, but still developed a pseudo tumor. 

Metal hypersensitivity as a reason for failed MoM prostheses has been reported [25,32]. Metal hypersensitivity may be a pre-existing condition or be due to metal exposure from MoM implants. Schalock *et al.* [33] presented an algorithm to diagnose metal hypersensitivity, suggesting doing patch test pre-operative in patients with suspected allergy (e.g., dermatitis) and post-operative in case of a suspected metal allergy. The recommendation, however, is that preoperative patch testing and lymphocyte transformation test (LTT) should only used in selected patients [34]. 

## 4. Conclusions 

**Figure 5:**
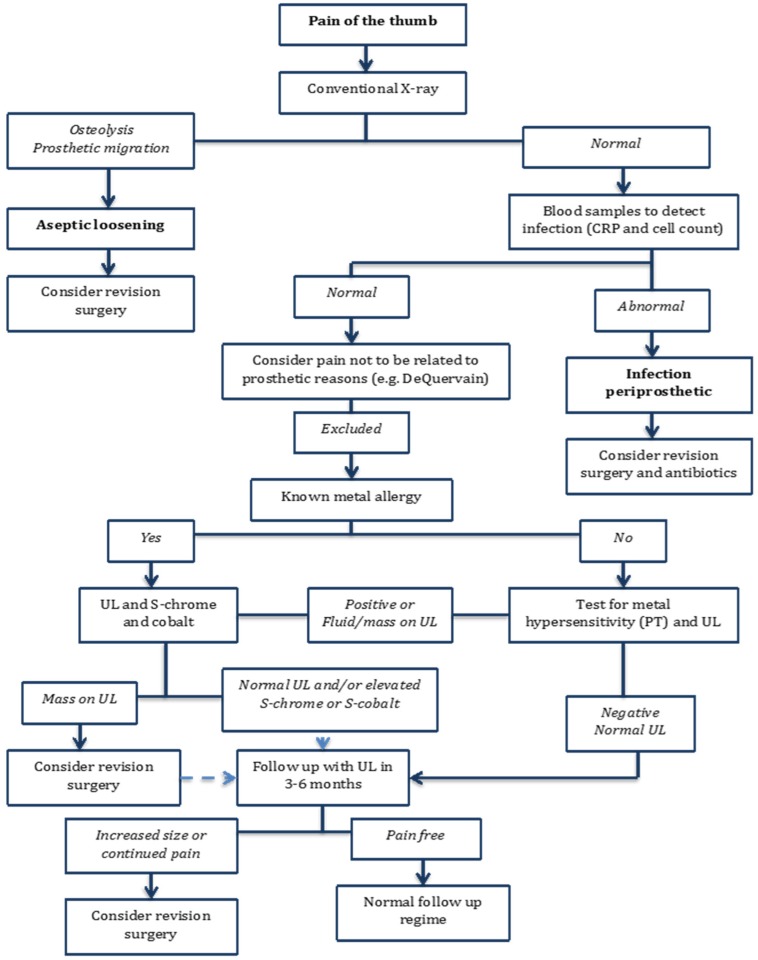
Diagnostic algorithm to the patient presenting with a painful thumb following surgery with metal-on-metal (MoM) trapeziometacarpal arthroplasty.

Based on the experiences from complications in MoM total joint hip arthroplasty we suggest a diagnostic algorithm (Figure 5) in the patient presenting with a painful thumb following surgery with MoM trapeziometacarpal arthroplasty. 

At first infection and aseptic loosening should be ruled out starting with a radiological examination. Osteolysis or migration of the prosthesis on plain radiographs should lead to explorative surgery in the patient with a painful joint, and material from the revision surgery should be sent to culture and microscopy to reveal any infection or pseudo tumor mass. Radiographs without signs of implant migration or osteolysis should lead to blood samples to rule out infection, however it should be taken into consideration, that adverse reactions to metal debris may simulate infection. When infection has been ruled out, other common reasons for thumb pain should be considered, e.g., De Quervain syndrome, but if no other explanation is found a MoM related failure should be considered. Questioning the patient for known allergies to metal and on suspicion a test for metal hypersensitivity with patch testing should be performed. Furthermore an ultrasound should be performed to reveal any fluid collection or mass surrounding the prosthesis. We recommend ultrasound in the initial screening for pseudo tumors as it is as sensitive as MRI, but more accessible and there are no contraindications [35]. If ultrasound reveals a mass surrounding a pseudo tumor must be suspected, and revision surgery should be considered. If no mass is found or only fluid collection is seen at the ultrasound a follow up visit in 3 months should be scheduled with a new ultrasound examination. 

Serum metal ion levels should be obtained. No clear recommendations for acceptable values for serum-cobalt and serum–chromium have been established, but The Medicines and Healthcare Products Regulatory Agency (MHRA) in the UK has set a upper limit at 7 parts per billion [36]. A patient with elevated metal ion levels and a normal ultrasound should be re-evaluated after 3–6 months with a new ultrasound. At follow up an increased fluid collection or new findings on ultrasound with persistent pain should give consideration to perform a revision surgery. Material from the surgery should be sent to cultures and histology to determine the origin. 

This algorithm is primarily based on the experiences published in articles concerning MoM hip arthroplasty and may not be directly transferred to trapeziometacarpal arthroplasties. However we suggests that pseudo tumor formation may be a generalized problem in MoM arthroplasties and find that the experiences from hip arthroplasties is at present the best evidence in treating MoM complications with metal hypersensitivity or pseudo tumor formation in trapeziometacarpal MoM total joint arthroplasty.
